# The influence of candidates’ physical attributes on patient ratings in simulated assessments of clinical practice

**DOI:** 10.1080/0142159X.2022.2093177

**Published:** 2022-07-12

**Authors:** C. A. Brown, K. Badger, M. D. Reid, R. Westacott, M. Gurnell, M. W. R. Reed, G. Chamberlain, E. Hatfield, A. Sharif, A. H. Sam

**Affiliations:** a Division of Health Sciences, Warwick Medical School, Coventry, UK; b Imperial College School of Medicine, Imperial College London, London, UK; c Birmingham Medical School, University of Birmingham, Birmingham, UK; d Wellcome–MRC Institute of Metabolic Science, University of Cambridge and NIHR Cambridge Biomedical Research Centre, Cambridge University Hospitals, Cambridge, UK; e Brighton and Sussex Medical School, University of Sussex, Brighton, UK

**Keywords:** Assessment, medicine, clinical

## Abstract

**Background:**

We have previously shown that clinical examiners’ scoring is not negatively impacted when a candidate has a tattoo, unnatural hair colour, or a regional accent. We investigated whether these physical attributes in exam candidates impact patient scoring.

**Methods:**

Simulated/real patients were randomly assigned to watch five videos of simulated candidate performances of a cranial nerve examination: clear fail, borderline, good, ‘clear pass’ without an attribute, and ‘clear pass’ with one of the attributes (tattoo, purple hair, accent). Participants scored domains of communication and professionalism. We compared scores for the clear pass candidates with and without attributes.

**Results:**

One hundred and eighty three patients participated. The total scores for the candidates with tattoos and purple hair were higher than the candidate with no physical attribute (*p* < 0.001). For the candidate with a Liverpool English accent no difference was identified (*p* = 0.120).

**Conclusions:**

The presence of certain physical attributes (tattoos or purple hair) was associated with higher scores given by patients to candidates in a simulated physical examination station.

Practice point

- Scores awarded by patients to candidates in medical assessments may be influenced by the presence of certain physical attributes.

## Introduction

Assessments based on observations of clinical skills, including history taking and physical examination, are integral to the assessment of clinician competence and often contribute to high-stakes assessments. Currently in the UK there is no standard approach to clinical skills assessment and as such there is significant institutional variation (MacDougall 2015). However, in 2024 the UK Medical Licensing Assessment (UKMLA) will be introduced. This will combine an Applied Knowledge Test (AKT) with a Clinical and Professional Skills Assessment (CPSA) to ensure those who obtain registration with a licence to practise medicine in the UK meet a common threshold for safe practice. It is therefore important to begin to explore those variables that could potentially contribute to variance in such high stakes assessments.

The use of professional simulated patients (SPs), who are trained to accurately and consistently portray a role, and ‘real’ volunteer patients (VPs), who usually have a condition or clinical sign that a newly graduating junior doctor would be expected to be familiar with, is a key aspect of clinical skills assessment. However, the extent to which simulated patient scoring of candidates is incorporated in assessments is variable and an array of rating tools are in use. Importantly, patient ratings have been shown to differ from those of clinicians, and it has been suggested that these reflect valid differences in perspective, which can enrich student assessment (Thistlethwaite 2004). Assessments that use simulated patients have been found to reliably and validly measure aspects of professional behaviour and communication skills (van Zanten et al. 2005; Weidner et al. 2010). Inclusion of one or more measures of patient experience demonstrates that assessors value the patient voice and is in accordance with the shift to clinical care models where patients are seen as key stakeholders in their healthcare (Wallace et al. 2002).

As with examiner scoring, an individual patient’s assessment of an exam candidate may theoretically be affected by several sources of rater errors and be subject to stereotype biasing. Interestingly, previous research has yielded mixed findings, with some studies suggesting physical attributes such as ethnicity can impact attainment in clinical exams whilst others have not shown differences (Schleicher et al. 2017; Yeates et al. 2017; Sam et al. 2021b). However, within society it is evident that stereotype biases do exist and these have been shown to impact patient perceptions within healthcare. For example, extravagant hair colour (Yonekura et al. 2013) or tattoos (Baumann et al. 2016; Broussard and Harton 2018) have been shown to negatively impact patient perspectives in a healthcare setting. In addition, people with a Liverpool English accent have been perceived as less trustworthy than those with a Standard Southern British English (SSBE) accent (Torre et al. 2018). Importantly, previous research in this area has largely focussed on patient scoring during in-person assessment, when there is the possibility of an audience effect whereby those present may facilitate or inhibit performance (Chen et al. 2019). Here, we use a video-based design to mitigate this source of bias.

Understanding whether candidates’ physical attributes can impact the scores they are awarded by simulated patients during high stakes clinical examinations (and lead to differential attainment) is therefore important. To address this, we developed a study in which standardised videos that had previously been validated in a study of clinical examiners (Sam et al. 2021a, 2021b) were individually scored by a group of simulated and real patient volunteers to determine if physical attributes influenced the scores which were awarded for different candidates who were performing at comparable levels.

## Methods

### Study design

A single-blinded, video-based, experimental, randomised, internet-based design.

### Procedure

**Figure 1.**
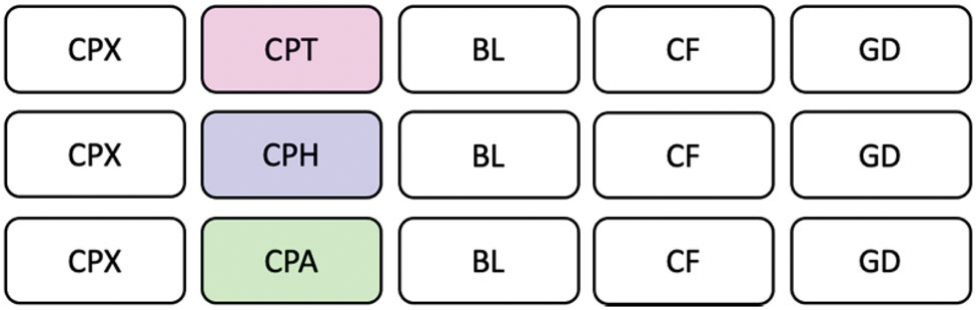
3 Master video sets viewed by 3 groups of participants: group 1 watched the master video set outlined in the first row (*n* = 59), group 2 watched the master video set outlined in the second row (*n* = 63) and group 3 watched the master video set outlined in the third row (*n* = 61). CPX: clear pass with no discernible attribute; CPT: clear pass with tattoos; CPH: clear pass with purple hair; CPA: clear pass with an accent; BL: borderline; CF: clear fail; GD: good.

Seven 10 minute videos were created of simulated candidates completing a cranial nerve examination. The same simulated patient was used in all videos to ensure standardisation. There was no examiner visible in the videos and the same voiceover was edited into the video to provide required examiner instructions. The simulated candidates were volunteer Clinical Teaching Fellows working with Imperial College London and all are White females of similar ages. Four of the videos demonstrated the simulated candidates performing the examination at the ‘clear fail’ (CF), ‘borderline’ (BL), ‘clear pass’ (CPX) or ‘good’ (GD) performance standard. The other three videos showed a candidate performing at a ‘clear pass’ level with either purple hair (CPH), tattoos (CPT), or a Liverpool English accent (CPA). The simulated candidates in all videos except CPA performed with a SSBE accent. The simulated candidates in all videos except CPH had either brown or blonde hair. Each candidate followed a script created by a panel of experienced examiners to ensure they were performing at the appropriate level and to standardise those performing at the ‘clear pass’ level. Three master sets of five videos were then created; with every set including a video of a candidate performing at each of the overall performance levels as well as one video of a candidate with a physical attribute performing at a ‘clear pass’ level (Figure 1). To minimise the impact of ordering effects on scoring from these three master video sets, a final total of 12 different video sets were created. The ordering of the five videos differed across the 12 sets and each participant was randomly allocated to one of the 12 video sets (Supplementary Appendix 1).

### Recruitment and consent

The study was approved by the Education Ethics Review Process at Imperial College London (EERP2021-011). Participants were informed that they were taking part in a study exploring inter-rater reliability amongst patient assessors but were not informed that the study aimed to evaluate the impact of physical attributes on scores and performance levels. Demographic data about the participants but no identifiable information was collected. Participants were required to be volunteer patients (VPs) or simulated patients (SPs) with previous experience in medical assessments or teaching. Participants were informed that completion of the marksheets for all five videos and submission of the post-completion questionnaire was evidence of consent. Participants were able to withdraw from the process by closing the web browser at any time prior to completion of the study but due to the lack of collection of identifiable data, were not able to withdraw after submitting their results. Any incomplete data was not used in the analysis.

### Patient measures

**Figure 2.**
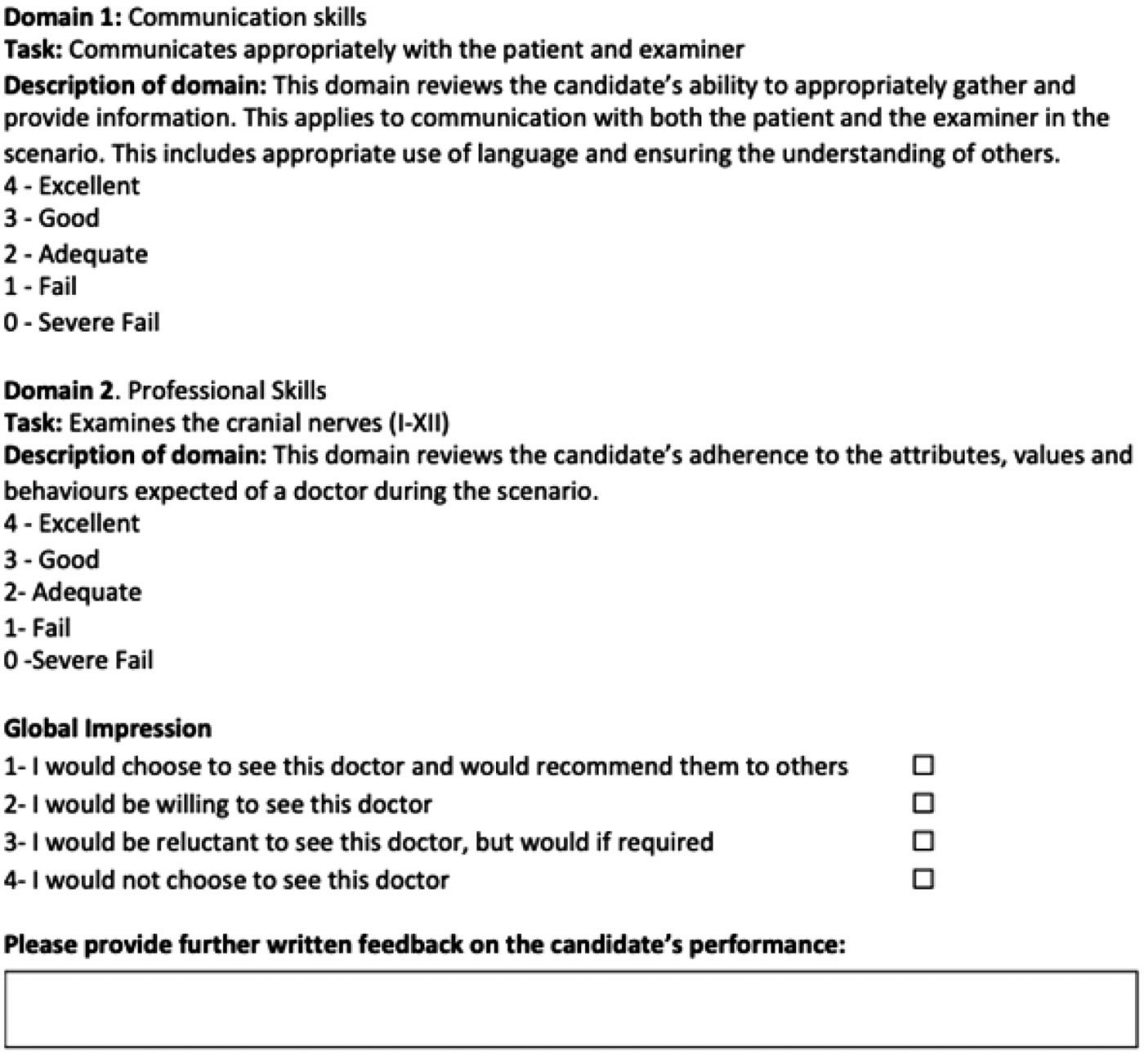
Sample mark sheet.

Participants were asked to assess the candidates at the level expected of a newly qualified doctor (Foundation Year 1 Doctor). Participants viewed the five videos and completed an online marksheet contemporaneously (Figure 2). Patients marked each candidate in two domains; ‘Communication skills’ and ‘Professional skills.’ Both domains were scored between 0 and 4, with a maximum possible total score of 8. Participants were also asked to assign each candidate a global impression: ‘I would not choose to see this doctor,’ ‘I would be reluctant to see this doctor, but would if required,’ ‘I would be willing to see this doctor’ or ‘I would choose to see this doctor and would recommend them to others.’ Finally they were asked to ‘Please provide further written feedback on the candidate’s performance’ via a free text box. The participant information sheet outlined to participants that they were able to return to mark sheets for previous candidates but were not able to pause, rewind or replay the videos, to reflect marking in exam conditions. Following completion of the mark sheets for all five videos, participants were asked to provide demographic details including whether they were a professional patient actor or a volunteer patient, gender, ethnicity, the geographical region where they worked and their associated medical school. They were also asked to quantify their previous assessment and teaching experience.

### Statistical analysis

Data management and analysis were conducted using Stata V16. For each candidate a total score from 0 to 8 was calculated by summing the two domain scores of communication and professional skills. For each participant, the difference between the total score awarded to CPX and the total score of the clear pass candidate with an attribute [CP(H/T/A)] was calculated. The median difference across participants, interquartile range of this difference and Wilcoxon matched-pairs signed-rank tests were used to compare the total scores for each attribute individually. The percentage agreement in global impressions for each candidate with an attribute was calculated by the number of participants who rated the candidate with the attribute at the same global grade which they gave the CPX candidate.

## Results

### Participants

Two hundred and forty three patients participated in the study of whom one hundred and eighty three were included in the analysis (sixty participants did not complete viewing and rating all of the candidates). Table 1 shows the demographic details of all participants included in the analysis. Participants included in the study have been involved in medical education at 35 different UK medical schools, as volunteer patients (VPs), most of whom were recruited due to the presence of a clinical condition of interest to medical student learning or as professional actors who work as simulated patients (SPs).

**Table 1.** Demographic Details of Patient Participants according to video set viewed.

		CPH	CPT	CPA
	*n* (%)	*n* (%)	*n* (%)	*n* (%)
	183 (100.0%)	63 (34.4%)	59 (32.2%)	61 (33.3%)
Role				
Professional Simulated Patient (SP)	143 (78.1%)	49 (77.8%)	49 (83.1%)	45 (73.8%)
Volunteer Patient (VP)	40 (21.9%)	14 (22.2%)	10 (16.9%)	16 (26.2%)
Gender				
Male	95 (51.9%)	35 (55.6%)	28 (47.5%)	32 (52.5%)
Female	85 (46.4%)	27 (42.9%)	31 (52.5%)	27 (44.3%)
Prefer not to say	3 (1.6%)	1 (1.6%)	0 (0.0%)	1 (1.6%)
Assessment Experience				
None	3 (1.6%)	0 (0.0%)	1 (1.7%)	2 (3.3%)
1–2 exams	10 (5.5%)	1 (1.6%)	4 (6.8%)	5 (8.2%)
3–4 exams	19 (10.4%)	7 (11.1%)	5 (8.5%)	7 (11.5%)
5+ exams	150 (82.0%)	55 (87.3%)	49 (83.1%)	46 (75.4%)
Prefer not to say	1 (0.5%)	0 (0.0%)	0 (0.0%)	1 (1.6%)
Teaching Experience				
None	34 (18.6%)	12 (19.0%)	10 (16.9%)	12 (19.7%)
1–2 sessions	10 (5.5%)	1 (1.6%)	4 (6.8%)	5 (8.2%)
3–4 sessions	7 (3.8%)	1 (1.6%)	5 (8.5%)	1 (1.6%)
5–10 sessions	17 (9.3%)	7 (11.1%)	5 (8.5%)	5 (8.2%)
10+ sessions	110 (60.1%)	40 (63.5%)	34 (57.6%)	36 (59.0%)
Prefer not to say	5 (2.7%)	2 (3.2%)	0 (0.0%)	2 (3.3%)

CPH: Video set containing ‘clear pass’ candidate with purple hair; CPT: Video set containing ‘clear pass’ candidate with tattoos; CPA: Video set containing ‘clear pass’ candidate with an accent.

### Total scores

Total scores, the sum of communication and professional skills domain scores which excludes the Global Impression Grade, for all four clear pass candidates (CPX, CPH, CPT and CPA) ranged from 2 to 8. The median and inter-quartile range (IQR) scores for each candidate were as follows: CPX median score 6, IQR 6–8; CPH median score 8, IQR 6–8; CPT median score 8, IQR 7–8; CPA median score 7, IQR 6–8. Individual Wilcoxon matched-pairs signed-ranks tests were performed on the total scores for the clear pass candidate with no attribute when compared to each clear pass candidate with an attribute. For the candidate with purple hair (CPH) and for the candidate with tattoos (CPT) this indicated that scores were statistically significantly higher than the candidate with no stereotypical attribute (CPX) (Table 2). For the candidate with a Liverpool English Accent there was no statistically significant difference (Table 2).

**Table 2.** Difference between CPX Total Score and each attribute (CPH, CPA and CPT) total score.

Difference = (CPX – CP(H/P/T))	*n*	Median	IQR	Wilcoxon Matched Pairs	
				*z*-value	*p*-value
CPH	63	0	–2 to 0	–4.181	<0.001
CPA	61	0	–1 to 0	–1.565	0.120
CPT	59	–1	–2 to 0	–3.873	<0.001

Negative values of median difference indicate that the candidate with the attribute has a higher total score than the candidate without the attribute.

### Global impressions

The global impression for the four clear pass candidates (CPX, CPH, CPT and CPA) varied from ‘I would not choose to see this doctor’ to ‘I would choose to see this doctor and would recommend them to others.’ For CPH, 55.6% of patients gave the same global impression rating as that given to CPX, 30.2% gave a higher rating and 14.2% gave a lower rating. For CPA 58.7% of patients gave the same global impression rating as that given to CPX, 17.5% gave a higher rating and 23.8% gave a lower rating. For CPT 54.3% gave the same global impression rating as that given to CPX, 33.9% gave a higher rating and 11.8% gave a lower rating. Therefore for both CPH and CPT, patients more commonly gave a higher global impression score than to CPX. For CPA, patients more commonly gave a lower global impression score than to CPX. Table 3 outlines the global impression scoring between CPX and CP(H/A/T).

**Table 3.** Global impression scoring awarded to candidate with no clear attribute (CPX) and the candidates with purple hair (CPH), tattoos (CPT) and an accent (CPA) by each patient participant (*n* (%)).

	CPX				
	Global Impression *n* (%)	‘I would not choose to see this doctor’	‘I would be reluctant to see this doctor, but would if required’	‘I would be willing to see this doctor’	‘I would choose to see this doctor and would recommend them to others’
CPH	‘I would not choose to see this doctor’	0 (0.0)	0 (0.0)	0 (0.0)	0 (0.0)
	‘I would be reluctant to see this doctor, but would if required’	0 (0.0)	0 (0.0)	0 (0.0)	0 (0.0)
	‘I would be willing to see this doctor’	1 (1.6)	1 (1.6)	8 (12.7)	7 (11.1)
	‘I would choose to see this doctor and would recommend them to others’	0 (0.0)	1 (1.6)	18 (28.6)	27 (42.9)
CPA	‘I would not choose to see this doctor’	0 (0.0)	0 (0.0)	0 (0.0)	0 (0.0)
	‘I would be reluctant to see this doctor, but would if required’	0 (0.0)	0 (0.0)	2 (3.3)	3 (4.9)
	‘I would be willing to see this doctor’	0 (0.0)	1 (1.6)	16 (26.2)	9 (14.8)
	‘I would choose to see this doctor and would recommend them to others’	0 (0.0)	3 (4.9)	8 (13.1)	21 (34.4)
CPT	‘I would not choose to see this doctor’	0 (0.0)	0 (0.0)	0 (0.0)	0 (0.0)
	‘I would be reluctant to see this doctor, but would if required’	0 (0.0)	0 (0.0)	0 (0.0)	1 (1.7)
	‘I would be willing to see this doctor’	0 (0.0)	0 (0.0)	6 (10.2)	6 (10.2)
	‘I would choose to see this doctor and would recommend them to others’	0 (0.0)	1 (1.7)	19 (32.2)	26 (44.1)

## Discussion

We set out to determine whether the presence of certain physical attributes might adversely impact scores awarded to examination candidates by simulated or real patients. However, perhaps unexpectedly, our study showed that patients gave higher scores to candidates with either purple hair or tattoos than to the candidate with no discernible physical attribute. There was no statistically significant difference between the candidate with a Liverpool accent and the candidate with no discernible physical attribute. These findings overlap with those of our previous study which showed that amongst examiners the presence of purple hair colour resulted in higher scores, but the presence of tattoos or a Liverpudlian accent resulted in no significant differences (Sam et al. 2021b). Whilst it is reassuring that any potential assessor biases did not appear to translate into a negative impact on candidates’ scores, our findings suggest that the presence of a notable characteristic may lead to higher scores possibly as the candidate stands out. In relation to the tattooed candidate these findings are novel, as previous research has identified either no discernible difference or negative impacts on patient perceptions (Baumann et al. 2016; Broussard and Harton 2018; Cohen et al. 2018). Additionally, whilst higher scores were demonstrated for the candidate with purple hair both in this study and a previous study looking at medical examiners (Sam et al. 2021b), previous research has demonstrated that extravagant hair colours impact negatively on patient perceptions (Yonekura et al. 2013). Therefore while these findings are important in developing understanding about the impact of bias on scoring in medical assessments, it perhaps highlights the unpredictability of the impact of stereotype bias. As stereotypes are linked to social context, these findings may relate to changing societal norms or cultural differences between two different countries (UK and Brazil). For example the increased acceptability of tattoos identified within our study may relate to increasing numbers of people with tattoos, but this requires further investigation (Spears and Manstead 1989).

### Limitations

This study used video recordings of simulated candidates performing a clinical examination, and therefore findings may not be generalisable to other assessment formats (Of note, it would be interesting to analyse the impact within the context of a clinical history station where interpretation of professional and communication skills may differ). Furthermore, the transferability of our findings to an in person (face-to-face) assessment may be limited as the videos used in the study only provided a single lens viewpoint and did not include examiner interactions with candidates. For participants it is possible that social desirability bias has created artificial inflation in the ratings as participants may have been consciously attempting to remain ‘fair’ in their scoring. Additionally, despite efforts to control other sources of variability amongst the actors used, it is possible that some of the observed effects resulted from variations between candidates. As participation was voluntary, self-selection bias may mean results are not generalisable to the entire patient population involved in medical education. There may also have been demographic factors in the participants that were not identified (such as age/gender/shared characteristics with candidates in the videos) that impacted on outcomes, but this study was not powered to analyse this degree of granularity. Importantly, this study compares the impact of bias on candidates performing at a ‘clear pass’ standard, and therefore the impact of bias on candidates performing at different standards may vary from our findings. We also recognise the impact that mark schemes may have on outcomes and our results may therefore not be generalisable if significantly different scoring rubrics are used. It is also important to acknowledge that due to a low number of volunteer patient participants the study was not powered to enable a meaningful comparison between the volunteer and professional simulated patient populations. Finally, this study was completed at a single time point, but any systematic effect of bias based on stereotype activation may vary over time as societal attitudes towards individual attributes also change.

### Future work

Further work is required to explore these findings, including analysis of effects in the real-world setting, assessing other clinical domains such as history taking, in other types of assessment, in the clinical context with workplace-based assessment and with other physical attributes. The role of both simulated and real patient scoring in assessment requires further research as we move towards patient-centred education and consider a national clinical assessment in the United Kingdom.

## Supplementary Material

Supplemental Material
