# Supplementary material for: The influence of candidates’ physical attributes on patient ratings in simulated assessments of clinical practice
Source: Med Teach. 2022 Jul 12;44(11):1277–82. doi: 10.1080/0142159X.2022.2093177 (PMC9612930; doi:10.1080/0142159X.2022.2093177)
Supplement: Supplemental Material [file IMTE_A_2093177_SM4606.docx]

**Appendix 1 – Video ordering**

| **Version** | **Video 1** | **Video 2** | **Video 3** | **Video 4** | **Video 5** |
| --- | --- | --- | --- | --- | --- |
| 1 | CPX | CPH | BL | CF | GD |
| 2 | CPX | CF | CPH | GD | BL |
| 3 | CPX | BL | GD | CPH | CF |
| 4 | CPX | GD | CF | BL | CPH |
| 5 | CPX | CPT | BL | CF | GD |
| 6 | CPX | CF | CPT | GD | BL |
| 7 | CPX | BL | GD | CPT | CF |
| 8 | CPX | GD | CF | BL | CPT |
| 9 | CPX | CPA | BL | CF | GD |
| 10 | CPX | CF | CPA | GD | BL |
| 11 | CPX | BL | GD | CPA | CF |
| 12 | CPX | GD | CF | BL | CPA |

**Key**: CF = clear fail, BL = borderline, GD = good, CPX = clear pass with no discernible attribute, CPH = clear pass with purple hair, CPT = clear pass with tattoos, CPA = clear pass with an accent
